# Dietary Sodium and Other Nutrient Intakes among Patients Undergoing Hemodialysis in New Zealand

**DOI:** 10.3390/nu10040502

**Published:** 2018-04-18

**Authors:** Zhengxiu Xie, Rachael McLean, Mark Marshall

**Affiliations:** 1 Department of Human Nutrition, University of Otago, Dunedin 9054, New Zealand; solid.dietitian@gmail.com; 2 Department of Preventive and Social Medicine, Dunedin School of Medicine, University of Otago, Dunedin 9054, New Zealand; 3 Department of Renal Medicine, School of Medicine, University of Auckland, Auckland 1023, New Zealand; markrogermarshall@icloud.com; 4 Department of Renal Medicine, Counties Manukau District Health Board, Auckland 2025, New Zealand; 5 Baxter Healthcare (Asia) Pte Ltd., Singapore 189720, Singapore

**Keywords:** renal dialysis, sodium dietary, diet, energy intake, New Zealand

## Abstract

This study describes baseline intakes of sodium and other nutrients in a multi-ethnic sample of hemodialysis patients in New Zealand participating in the SoLID Trial between May/2013 to May/2016. Baseline 3-day weighed food record collections were analyzed using Foodworks 8 Professional food composition database, supplemented by other sources of nutrient information. Intakes of dietary sodium and other nutrients were compared with relevant guidelines and clinical recommendations. Eighty-five participants completed a 3-day weighed food record. The mean (SD) sodium intake was 2502 (957) mg/day at and more than half of the participants exceeded recommended intake levels. Sodium intake was positively associated with energy intake. Only 5% of participants met the recommended calorie density; nine percent of participants ate the recommended minimum of 1.2 g/kg of protein per day; 68% of participants were consuming inadequate fiber at baseline. A high proportion of dialysis patients in SoLID Trial did not meet current renal-specific dietary recommendations. The data show excess sodium intake. It is also evident that there was poor adherence to dietary guidelines for a range of other nutrients. A total diet approach is needed to lower sodium intake and improve total diet quality among hemodialysis patients in New Zealand.

## 1. Introduction

Chronic kidney disease is associated with increased risk of mortality, cardiovascular disease (CVD) (including coronary heart disease and stroke) [1]. The global burden of disease study showed that from 1990 to 2010, CKD had risen from the 27th to the 18th leading causes of death globally [2]. The prevalence of dialysis is increasing especially amongst vulnerable populations, such as indigenous and those of low socioeconomic status. The survival of patients on dialysis is poor and limited by the development or exacerbation of cardiovascular disease, protein-calorie malnutrition, and immune dysregulation [1]. There are approximately 2.5 million patients worldwide undergoing dialysis treatment with 1859 undergoing hemodialysis in New Zealand in 2014. The prevalence of dialysis treatment in New Zealand is increasing, with disproportionate growth relative to the general population, and over-representation of Māori and Pacific people in the dialysis population [3]. 

Dietary therapy plays a critical role in kidney health, where impaired excretion of waste and altered metabolism interferes with normal homeostasis. Hypertension is present in most dialysis patients, and attributed to fluid overload and neurohormonal factors, both of which will be affected by dietary sodium intake [4,5]. Recent international data on sodium intake in dialysis patients reveals that there is a wide range of sodium intake from 1520 mg to 9540 mg/day [6,7,8]. Current guidelines recommend in the international literature recommend that dialysis patients consume 1500–3000 mg/day of sodium [9,10,11,12,13,14,15,16,17].

Dietary sodium intake, however, is intrinsically linked with other nutrient intake, and cannot be considered in isolation—sodium intake is most relevant when considered within the broader context of the patients’ overall dietary intake.

The current sodium intake of hemodialysis (HD) patients is deserving of further study. Because of the importance of overall diet quality, this paper describes baseline dietary intakes of sodium and other nutrients in a multicenter, cohort of hemodialysis patients participating in the Sodium Lowering in Dialysate (SoLID) Trial (ACTRN12611000975998), which was conducted in New Zealand to evaluate the use of low sodium dialysate in HD patients.

## 2. Materials and Methods

This descriptive study describes baseline intakes of sodium and other nutrients based on 3-day weighed food records. Data are sourced from the SoLID Trial, a completed multi-center prospective, single-blind, randomized controlled, clinical trial of low (135 mmol/L) versus conventional (140 mmol/L) (Na^+^) dialysate during HD. A full description of study protocol can be found elsewhere (www.solid.org.nz) [18,19,20].

Participants were patients on hemodialysis recruited in 10 centers from 7 District Health Boards (DHBs) throughout New Zealand (Counties Manukau, Auckland, Waitemata, Waikato, Capital & Coast, Canterbury, and Southern), with an accrual period of 36 months between May 2013 to May 2016, and patient follow-up of 12 months. Participants were eligible for the study if they were incident or prevalent patients treated with maintenance home or self-care satellite hemodialysis for end-stage kidney disease, aged 18 years or older, suitable for both low and standard (Na^+^) dialysate in the view of the treating physician, and able to give informed consent to participate in the study. The following participants were excluded: those undergoing hemodialysis treatments at a frequency of greater than 3.5 times per week; those receiving treatment with maintenance hemodiafiltration; those expected to be in the study for less than 12 months because of either poor life expectancy or expected renal transplantation; those with concomitant illness or health conditions that limiting or contraindicating study procedures and follow-up; those with a high chance of non-adherence; and those currently involved in other clinical trials of antihypertensive medications or hemodialysis regimen.

The SoLID Trial received the National (New Zealand) multi-region ethical approval, and institutional approval in each of the centers that were involved in the SoLID Trial (approval number: MEC/11/09/081). Written consent was obtained from all patients agreeing to participate. Patients who were ineligible or who declined to participate continued with usual care, which is reviewed by renal dietitian every 6 months or more often on request.

We report on sociodemographic, clinical and dietary assessments from the baseline assessment in the SoLID trial, which was performed prior to randomization. 

Dietary variables include daily intake of sodium, energy, total fat, saturated fat, carbohydrate, phosphate, calcium, potassium and fiber; density of calories and protein-intake as per Kg of body weight; dietary sodium to potassium molar ratio.

Given our primary focus on dietary sodium intake, study findings are presented in categories of dietary sodium intake: low sodium intake (<1840 mg/day), recommended sodium intake (1840–2300 mg/day), and high sodium intake (>2300 mg/day) according to evidence based practice guidelines for nutritional management of chronic kidney disease in New Zealand [9].

Weight was measured either on the scales in the dialysis unit or on the patient scales at home, and recorded before they dialyzed, after their long break if they dialyzed three times per week or mid-week if they dialyzed on alternate days. Patients were asked to remove anything from their pockets prior to weighing. Height was measured either at the dialysis unit or taken from clinical notes.

Blood pressure was taken using an Omron electronic sphygmomanometer (https://omronhealthcare.com/), in the sitting position. Blood pressure was recorded just before they dialyzed after long dialysis break or mid-week if they dialyzed alternate days. A minimum of four readings (wherever possible) were taken, and the mean of these measures was used for this analysis.

For dietary assessment, each participant was asked to record a 3-day weighed food diary at home at baseline. The research coordinator delivered standard 3-day food recording sheets with detailed participant instructions, Salter digital scales, and standard measuring cups/spoons. Participants were asked to record everything they consumed on each of the three recording days, including the quantity of the food, brand of any processed foods consumed, or details of recipes used for homemade foods. The protocol stated that the 3 recording days should include at least one-weekend day and one-week day, and also one dialysis day and one non-dialysis day. The 3-day weighed food diaries included a detailed assessment of how much added salt and water participants ingested, including drinks of water and salt added in cooking and at the table. 

Completed food diaries were reviewed for errors (e.g., implausible weights recorded such as kilograms being entered instead of grams), clarity, consistency, and completeness. The Research Dietitian contacted participants directly by telephone or email to confirm brand names, recipes, or measurements when recordings were unclear, inconsistent or incomplete. When the participant was not the one primarily responsible for cooking in the family, the person most responsible was also contacted. Once the review was completed, the Research Dietitian entered the details into Foodworks 8 professional (http://www.xyris.com.au/). Other sources of nutrients information for recorded food items were also used including Nutritrack fast food and supermarket database (www.nutriweb.org.nz) [21] as well as online databases and Original Equipment Manufacturer (OEM) packages sourced from local retail outlets.

Daily intakes were compared to relevant guidelines, using current New Zealand guidelines whenever possible. Recommended intakes for protein, sodium, phosphorus, fiber for HD patients were taken from the 2016 New Zealand Dietitian Clinical Handbook [9]; recommended energy intake from National Kidney Foundation (NKF) Kidney Disease Outcomes Quality Initiative (K/DOQI) guidelines [22]; recommended saturated fat intake from 2006 Australia and New Zealand Renal Group Taskforce Guidelines for CKD (10); recommended New Zealand healthy adult total fat and carbohydrate intakes (percentage of total energy) [23]; recommended potassium intake level from 2007 European Best Practice Guidelines on Nutrition for HD [11]; recommended sodium/potassium ratio from 2012 WHO Sodium Intake Guideline [24] As there is no specific calcium intake recommendation in HD patients, we used the reference value cited in Luis et al. [25].

### Statistical Analysis

Dietary intake was expressed as the intake per day of nutrients, averaged over the 3 days of food diaries. All analyses were undertaken using Stata (Version 12.1) [26]. Pearson’s correlation was undertaken between sodium intake and energy and potassium intakes. 

## 3. Results

In the original SoLID trial, six hundred and sixty-three patients were assessed for eligibility. One hundred and seventy-seven declined to participate, and 387 excluded due to not meeting inclusion criteria. Of the 99 participants recruited to the SoLID Trial, 85 completed a 3-day weighed food diary at baseline. 

Participant baseline characteristics are summarized in Table 1, which also shows demographic characteristics by category of sodium intake (low, recommended and high sodium intake). The population consisted of 85 patients, 66% of whom were male, with a mean (SD) age of 52 (13) years. The largest ethnic group was ‘New Zealand European and Others’ (45%) followed by Pacific (29%). One third of participants had a diagnosis of diabetes mellitus. There were no significant differences in participant characteristics by category of sodium intake. There was no significant difference in sodium intake or sodium intake category by ethnic group, however low numbers in ethnic groups other than New Zealand European and Others may explain this finding, and no further ethnic specific analysis was undertaken. Forty-nine participants provided interdialytic urine sample with a mean (SD) of 800 (827) mL/day and the mean (SD) sodium excretion from the urine sample was 59 (66) mmol/day. 

### Daily Intake of Nutrients

Intakes of macronutrients and micronutrients are presented in Table 2 by category of sodium intake: below, at and above recommended intake levels. There is evidence of a linear association between nutrient intake and category of sodium intake for energy, calorie density, and protein and carbohydrate intakes in g/day but not for % total energy. There was also evidence of a linear association between intake of fiber, phosphate and potassium and category of sodium intake (all *p* < 0.05). 

Table 3 shows mean(SD) intake and the number (%) of participants who are adherent to recommended intakes for all nutrients assessed. Mean (SD) sodium intake was 2502 (957) mg/day (2591 mg/day for men and 2330 mg/day for women). Only 24% of participants met the recommended level of sodium intake of 1840–2300 mg/day, with 54% exceeding the recommended sodium intake range.

Only 5% of participants met the recommended calorie density of 30–35 kcal/kg. Mean protein intake was generally lower than recommended. For protein density, 9% of participants consumed the recommended minimum of 1.2 g/kg of protein per day. Protein intake contributed around 18% of total energy. Saturated fat intake contributed a mean of 13% total energy intake, higher than recommended. The mean carbohydrate intake accounted for 47% of total energy (at the lower end of recommendations). About two thirds of participants were consuming inadequate fiber. 

Fifty-two percent of participants has excess phosphorus intake. Fifty-eight percent of participants had less than 500 mg of dietary calcium intake daily. Sixteen percent of participants consumed more potassium than recommended. 

**Figure 1:**
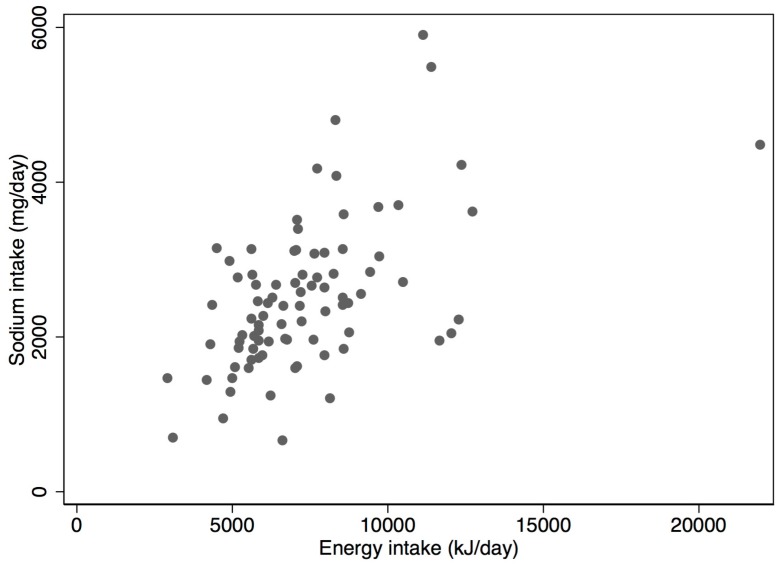
Scatter plot of energy and sodium intake for participants (*n* = 85).

Dietary sodium intake was statistically significantly and moderately correlated with energy intake (*r* = 0.55, *p* < 0.001) (Figure 1). Sodium intake was also positively correlated with potassium intake (*r* = 0.28, *p* = 0.009). 

## 4. Discussion

Although there have been no long-term randomized controlled trials of dietary sodium restriction in hemodialysis patients, available literature shows the benefits of dietary sodium restriction, in conjunction with fluid restriction. Positive associations include reduced inter-dialytic weight gain, reduced blood pressure, reduced left ventricular mass, inflammation, and perhaps even improved survival rates [27]. As a result, restricting sodium intake has become customary practice for HD patients around the world [28,29,30,31,32].

We found that few participants in the SoLID trial adhered to New Zealand guidelines for sodium intake, and that this was nested in a wider degree of dietary non-adherence involving all other nutrients studied. Mean sodium intake was 2502 mg/day (2591 mg/day for men and 2330 mg/day for women) above the recommendations for patients on hemodialysis which recommend an intake of 1840–2300 mg per day [9]. The excessive sodium intake was consistent with a previous study of home hemodialysis in New Zealand [33], many other hemodialysis and healthy populations around the world [25,34,35,36,37,38]. Such non-adherence in the dialysis literature seems to be rooted in undesirable food choices, such as higher sodium fast foods and convenient processed foods [39]. It has been suggested that this results from the need to accommodate lengthy treatments, and the fatigue that is often experienced after dialysis [40,41]. An overview of food diaries in our study showed that this was also the case for the SoLID participants, who consumed fast foods and convenience foods regularly (data not shown). It is also known that people with CKD have impaired taste sensitivity which may be a factor contributing to excess salt intake in HD patients [42]. Research shows a short period of salt restriction improves the recognition threshold for salt taste in CKD patients [43] which could be used in dietary management of HD patients. Of note, although sodium intake in our cohort of SoLID participants was higher than recommended, it was still lower than population estimates of intake among healthy adults in New Zealand. A random sample of healthy New Zealand adults aged 18 to 64 years had a mean 24 h sodium excretion of 3386 mg/day (95% CI 3221, 3551): 3865 mg/day for men and for 2934 mg/day women [44]. However, the difference in measurement tools may account for some of this difference, as dietary assessment methods have been shown to underestimate sodium intake, compared to the gold standard 24 h urinary assessment in previous studies [37].

There is evidence that the general food environment in New Zealand in not conducive to maintaining a low sodium diet. In a ‘Western’ style diet, processed food contributes to 75–80% of salt intake [45]. A recent study examined the sodium content of New Zealand processed food between 2003 and 2013 and found only slight reductions in sodium concentration and increases for some food categories since 2003 [46]. Thomson et al. found that processed food alone contributed around 1714–3040 mg/day of sodium intake among healthy New Zealand adults (≥19 years old) based on the New Zealand nutrition survey 24-h diet recall information [47,48]. 

We found a positive association between sodium intake and energy intake in our study, a finding previously reported in both healthy and hemodialysis patients [32]. We also found evidence of a linear association between intakes of a number of nutrients and category of sodium intake, notably energy, calorie density and protein and carbohydrate intakes in grams per day, but not as %total energy (Table 2). This shows that sodium is inherent in much of the food commonly consumed, and therefore closely associated with the amount of food consumed. Importantly, estimated calorie density in SoLID participants was well below recommendations, and median energy intakes (7196 kJ/day for males, 5855 kJ/day for females) well below that reported for the general population in New Zealand (10,380 kJ/day for males, 7448 kJ for females) [49]. This is not an unusual finding, and thought to be due to the overall poorer health status of HD patients, and their decreased appetite [50]. However, this is a critical issue since efforts to intensify sodium restriction of risks compromising energy intake further. A recent pilot study among 11 healthy New Zealand adults demonstrated that adherence to a low salt diet is achievable, however substantial changes to eating habits were required [51]. 

Protein-energy wasting is very common among hemodialysis patients due to poor appetite, inflammation and nutrients loss during dialysis [50], and a minimum calorie and protein intake is necessary to avoid a caloric deficit and malnourishment [52]. The poor adherence of SoLID participants to recommended levels of intake is consistent with other studies [25,33,40]. Our results show that New Zealand HD patients should be counselled to increase protein intake. However, care should be taken in implementing this recommendation since increased protein intake will also contribute to increased phosphate intake and possibly consequent vascular calcification. In the SoLID Trial, more than half of dialysis patients already exceeded recommended phosphate intake at baseline. A recent Australian study showed that well-nourished hemodialysis patients could in fact have a protein intake lower than the current recommendations, and clearly dietary recommendations should be individualized [53]. In recent years, renal dietitians have also recognized the role of easily absorbed phosphorus-containing food additives in processed food [35], and active efforts are made by New Zealand renal dietitians encouraging patients to read labels and identify phosphorus-containing food additives.

In contrast to protein intake, a relatively high proportion of patients in our study consumed relatively high total fat, often an important source of energy in dialysis patients. However levels of saturated fat were high at 13% of total energy intake. This intake of saturated fat is similar to that of the wider New Zealand population [49] and other dialysis patient population elsewhere [25,54]. Again, this may be related to choices of poor quality foods. Encouraging intake of lean meat/reduced fat dairy products while emphasizing sources of mono-unsaturated and polyunsaturated fatty acids may reduce cardiovascular disease risk [55]. By doing so, it will also help address issues around low protein intake. 

A number of barriers to adherence to a renal diet have been identified in international studies, using qualitative research methods. Morris et al. conducted semi-structured interviews with people on peritoneal dialysis in the United Kingdom and identified that eating outside the home was particularly problematic due to lack of control over food served, and reluctance to tell hosts of dietary restrictions for fear of judgement and attention from others [56]. Other research shows that cultural and environmental factors are important for dietary adherence to renal diets, particularly the requirement for detailed knowledge and peer and family support [57,58]. Individualised counselling and education are facilitators, as a renal diet is highly restrictive, and is different to what normally constitutes a ‘healthy diet’ particularly with respect to restricting fruit and vegetables [57]. Little is known however of barriers and facilitators to following a renal diet in a New Zealand context, and further research would be beneficial. 

There are several limitations to our study. First, we used a 3-day weighed food diary to assess sodium intake which is appropriate in this population where 24-h urine is not possible [37]. While acknowledging the limitations of food diaries, many strategies were used to ensure the best quality of 3-day weighed food diaries was obtained, for example, obtaining nutritional information from manufacturers’ websites and supermarkets, visiting cafes and personal email communications for recipes details, weighing food samples if weight was not provided by participants. Notwithstanding, the diaries were still self-reported, and we cannot exclude the presence of social desirability bias and underreporting [59,60]. Second, the food composition databases we used may not have always been accurate due to the ever-changing food market and the complexity of methods of cooking utilized by patients. Finally, the study population may not have been representative of the New Zealand hemodialysis population due to the eligibility criteria for the SoLID trial. 

## 5. Conclusions

The observed poor dietary quality and poor adherence to the current dietary recommendations in our study suggests that efforts are needed to intensify dietary intervention in hemodialysis patients in New Zealand. The particular challenge is to decrease sodium and phosphate intake without compromising energy and protein intake for better health outcomes. This requires minimizing processed foods and fast food and choosing lean low-fat protein food. Qualitative research is also required to enable a better understanding about barriers for the poor adherence to the dietary recommendations in a New Zealand context. Studies show poor knowledge and understanding about dietary recommendations among hemodialysis patients [61], but less is known about other barriers to achieving recommended nutrient intakes. A whole diet approach is needed rather than focus on individual nutrients in order to improve overall diet quality, to improve clinical outcomes.

## Figures and Tables

**Table 1:** Baseline characteristics of participants stratified according to categories of daily sodium intake based on New Zealand Guidelines.

Baseline Characteristics	All (*n* = 85)	Category 1 (Low Sodium Intake) <1840 mg/day (*n* = 19)	Category 2 (Recommended Sodium Intake) 1840–2300 mg/day * (*n* = 20)	Category 3 (High Sodium Intake) >2300 mg/day (*n* = 46)
Age, year mean (SD)	52 (13)	52 (12)	53 (14)	51 (13)
Men, *n* (%)	56 (66)	13 (68)	11 (55)	32 (70)
Women, *n* (%)	29 (34)	6 (32)	9 (45)	14 (30)
Ethnicity ^§^, *n* (%)				
NZEO **	38 (45)	6 (32)	9 (45)	23 (50)
NZ Maori	14 (17)	4 (21)	2 (10)	8 (17)
Pacific	25 (29)	8 (42)	8 (40)	9 (20)
Asian	8 (9)	1 (5)	1 (5)	6 (13)
Diabetes mellitus, *n* (%)	28 (33)	8 (42)	6 (30)	14 (30)
Dialysis, months mean (SD)	57 (57)	72 (75)	42 (36)	56 (54)
Weight, kg, mean (SD)	88 (20)	95 (28)	88 (19)	85 (16)
Height, m, mean (SD)	1.72 (0.1)	1.74 (0.11)	1.70 (0.1)	1.73 (0.09)
Body Mass Index (km/m^2^), mean (SD)	29.31 (6.88)	31.42 (8.7)	30.29 (5.49)	28.12 (6.42)
Systolic Blood Pressure, mmHg, mean (SD) ***	139 (21)	132 (26)	137 (20)	143 (17)
Diastolic Blood Pressure, mmHg, mean (SD) ***	78 (13)	75 (11)	75 (12)	81 (14)
Energy, kJ/day, mean (SD)	7376 (2645)	5811 (1556)	7047 (2347)	8165 (2836)
Hemoglobin, g/L	111 (15)	111 (15)	111 (15)	112 (15)
Serum creatinine, mmol/L	855 (281)	956 (252)	813 (177)	832 (322)
Serum urea, mmol/L	20.82 (8.21)	23.49 (9.40)	20.29 (5.88)	19.94 (8.49) ****
Adjusted Calcium, mmol/L	2.39 (0.2)	2.33 (0.28)	2.39 (0.16)	2.42 (0.17) ****
Serum phosphate, mmol/L	1.71 (0.52)	2 (0.56)	1.6 (0.41)	1.64 (0.5) ****
Serum albumin, g/L	38.35 (3.58)	37.63 (3.27)	38.25 (3.7)	38.7 (3.69)

^§^ Ethnicity was prioritized according to Statistics NZ standard. * Recommended level by NZ Dietitians Clinical handbook 2016. ** NZEO—New Zealand European and Others. *** Blood pressure data were missing for two patients. **** *n* = 45.

**Table 2:** Mean (SD) nutrient intakes, stratified according to categories of daily sodium intake based on New Zealand guidelines.

Nutrient Intakes	All (*n* = 85)	Category 1 (Low Sodium Intake) <1840 mg/day (*n* = 19)	Category 2 (Recommended Sodium Intake) 1840–2300 mg/day * (*n* = 20)	Category 3 (High Sodium Intake) >2300 mg/day (*n* = 46)	*p*
Energy, kJ/day	7376 (2645)	5811 (1556)	7048 (2347)	8166 (2836)	0.001
Calorie density, kcal/kg	20.96 (7.71)	15.91 (6.28)	19.67 (6.09)	23.6 (7.81)	<0.001
Protein, g/day	78 (26)	67 (20)	75 (29)	84 (25)	0.013
Protein, % TE	0.18 (0.04)	0.2 (0.04)	0.18 (0.04)	0.18 (0.04)	0.109
Protein density, g/kg	0.92 (0.32)	0.76 (0.28)	0.87 (0.33)	1.01 (0.31)	0.002
Total fat, g/day	70 (53)	75 (104)	62 (24)	71 (25)	0.914
Total fat, % TE	0.36 (0.3)	0.48 (0.61)	0.33 (0.08)	0.33 (0.07)	0.102
Saturated fat, % TE	0.13 (0.04)	0.13 (0.04)	0.13 (0.05)	0.13 (0.04)	0.703
Carbohydrate, g/day	204 (68)	163 (54)	205 (77)	220 (64)	0.002
Carbohydrate, % TE	0.47 (0.09)	0.47 (0.07)	0.48 (0.08)	0.46 (0.09)	0.748
Fiber, g/day	18.22 (7.34)	14.42 (6.23)	16.6 (5.57)	20.5 (7.71)	0.001
Phosphate, mg/day	982 (348)	885 (278)	849 (334)	1081 (355)	0.014
Potassium, mg/day	2146 (747)	1925 (735)	1899 (762)	2345 (701)	0.016
Calcium, mg/day	532 (705)	718 (1423)	396 (253)	515 (259)	0.416

* New Zealand Dietitians Clinical handbook 2016. % TE, % Total Energy.

**Table 3:** Daily intake of macronutrients and micronutrients and proportion of individuals within recommended targets.

Nutrients	Daily Intake Baseline (*n* = 85)	*n* (%) Within Target Values	Recommended Levels
Sodium, mg	2502	20 (24)	1840–2300 mg **
Energy, kJ	7376 (2645)	-	-
Calorie density, kcal/kg	20.96 (7.71)	4 (5)	30–35 kcal/kg **
Protein density g/kg	0.92 (0.32)	8(9)	1.2–1.4 g/kg **
Protein, % TE	18 (4)	-	-
Total fat, % TE	36 (30)	48 (56)	20–35% TE ***
Saturated fat, % TE	13 (4)	5 (6)	<7% TE ~
Carbohydrate, % TE	47 (9)	51 (60)	45–65% TE ***
Fiber, g	18 (7)	27 (32)	20–25 g **
Phosphorus, mg	982 (348)	14 (16)	800–1000 mg **
Calcium, mg	532 (705)	29 (34)	500–800 mg !
Potassium, mg	2146 (747)	40 (47)	1950–2730 mg #
Sodium potassium molar ratio	2.14 (0.94)	6 (7)	1:1 ^

% TE, Percent of total energy. ** Recommended levels by NZ Dietitians Clinical Handbook 2016. *** Recommended levels by Eating and Activity Guidelines for NZ Adults 2015. ~ Recommended level by the Australia and NZ Renal Guidelines Taskforce 2006. ! Cited in Luis et al., 2016 Journal of Renal Nutrition. # Recommended level by European Best Practice Guideline on Nutrition and Chronic Kidney Disease 2007. ^ Recommended level by WHO Guideline: Sodium Intake for Adults and Children 2012.
